# Deduction of probable events of lateral gene transfer through comparison of phylogenetic trees by recursive consolidation and rearrangement

**DOI:** 10.1186/1471-2148-5-27

**Published:** 2005-04-08

**Authors:** Dave MacLeod, Robert L Charlebois, Ford Doolittle, Eric Bapteste

**Affiliations:** 1 GenomeAtlantic, 1721 Lower Water Street, Suite 401, Halifax, NS, B3J 1S5, Canada; 2 Department of Biochemistry & Molecular Biology, Dalhousie University, 5850 College St., Halifax, NS, B3H 1X5, Canada

## Abstract

**Background:**

When organismal phylogenies based on sequences of single marker genes are poorly resolved, a logical approach is to add more markers, on the assumption that weak but congruent phylogenetic signal will be reinforced in such multigene trees. Such approaches are valid only when the several markers indeed have identical phylogenies, an issue which many multigene methods (such as the use of concatenated gene sequences or the assembly of supertrees) do not directly address. Indeed, even when the true history is a mixture of vertical descent for some genes and lateral gene transfer (LGT) for others, such methods produce unique topologies.

**Results:**

We have developed software that aims to extract evidence for vertical and lateral inheritance from a set of gene trees compared against an arbitrary reference tree. This evidence is then displayed as a synthesis showing support over the tree for vertical inheritance, overlaid with explicit lateral gene transfer (LGT) events inferred to have occurred over the history of the tree. Like splits-tree methods, one can thus identify nodes at which conflict occurs. Additionally one can make reasonable inferences about vertical and lateral signal, assigning putative donors and recipients.

**Conclusion:**

A tool such as ours can serve to explore the reticulated dimensionality of molecular evolution, by dissecting vertical and lateral inheritance at high resolution. By this, we mean that individual nodes can be examined not only for congruence, but also for coherence in light of LGT. We assert that our tools will facilitate the comparison of phylogenetic trees, and the interpretation of conflicting data.

## Background

### Phylogeny in a context of LGT

The Tree of Life has long been a central metaphor of evolutionary theory, and is a useful framework upon which to build a natural classification. However, understanding the phylogeny of species from gene trees is a very challenging task, both technically and conceptually.

First, not all genes are useful phylogenetic markers. Paralogs resulting from gene duplications occurring prior to the divergence of the organismal lineages being studied can easily confuse an analysis, as would happen, for instance, when unknowingly constructing a tree for mammals based on hemoglobin sequences from some species and myoglobins from others. For different reasons (see [1] for a detailed review), orthologs themselves must be scrutinized carefully. Notably, the phylogenetic signal in genes can be reduced by their limited size, radiative (star-like) evolution they may have undergone or the phenomenon of mutational saturation. Much of the time, the quality of phylogenetic signal is too low to resolve all of the problems under consideration in a single analysis. Multiple gene trees are often then exploited because interestingly, in each gene tree, some nodes may in fact receive significant support. Increasing the number of markers might then resolve the full tree, piecemeal. However, before accepting these individual relationships as solid, the phylogenetic quality and congruence between markers must also be tested.

Artifacts of tree reconstruction or the use of an inappropriate evolutionary model can lead to robust but artifactual groupings in gene trees. Recombination events producing mosaic genes can also blur phylogenetic trees and may produce controversial groupings. As a consequence, software has been developed to test that the signal carried by a single gene is not self-contradictory [2,3]. But even non-mosaic orthologs for different genes can be in conflict, when one of them has been laterally transferred.

This last source of conflicting signal is a major concern. All living systems from viruses [4] to eukaryotes [5] can participate in the transfer of genetic material. Lateral transfer occurs within domains of life, but also across domains, for different markers. There is now broad general agreement that lateral gene transfer (LGT) is a major force in the evolution of prokaryotes [5-7]. Additional evidence suggests that gene transfer might also be an important evolutionary mechanism in protist evolution [8]. For instance, Andersson *et al*. [9] recently reported that alanyl-tRNA synthetase had been transferred from Nanoarchaeota to Diplomonads and Parabasalids. The same authors [10] showed that LGT has affected both eukaryotes and prokaryotes with respect to glutamate dehydrogenase.

One may choose to ignore conflicting signal as if it were noise, even if legitimate evolutionary events underlie it. If such a choice is made, incompatibilities between different trees can be resolved by supertree [11-15] or *a posteriori *consensus approaches [16]. Supertree methods assemble an input set of separate phylogenetic trees with shared taxa into a larger tree [13,17] (or several trees). By fitting variously supported clades together, they allow large phylogenies based on different characters to be constructed rapidly and have been applied to a broad range of species [18]. Consensus approaches, such as obtained by concatenating sequences [19] or by averaging over a large number of genes [20] produce resolved phylogenies by overwhelming noise with signal that is presumed to be systematically congruent and historically true, though weak.

However, if this "noise" is in fact *bona fide *phylogenetic signal, then the tree cannot display a historical picture of evolution: internal nodes do not accurately represent the evolutionary process. It might be useful in taxonomy, indeed represent the best possible compromise for taxonomic purposes, but such a graph could do little to satisfy our understanding of molecular evolution. The averaging employed by these methods irons over what are perhaps the most interesting wrinkles.

However, it should still be possible to make progress towards the deeper goal of understanding genome and organism history, if we are willing to abandon the formalism of the tree, itself just a convenient metaphor for genealogical relationships. Horizontal inheritance, as complicated as it is to deal with within the constraints of the Tree paradigm, is simply a facet within a more realistic model of genealogy yet to develop fully. SplitsTree [2] and its kind are excellent at representing this higher dimensionality, though the webs that it draws are still just graphs of relationships. Little about evolutionary history can be gleaned from these graphs, though they do suggest compelling hypotheses.

Here, we are interested in retracing evolutionary history as much as possible, attending to both the vertical and horizontal axes of inheritance. We do not think that LGT should be dismissed as noise and discarded in the construction of a phylogeny, but neither do we wish to abandon the concept of vertical inheritance, which by the very nature of cell division, must be a factor in genealogy, overwhelmingly so in the short term. Instead of forcing each gene to fit a given model, therefore, our aim is to extract as much of a gene's phylogenetic information as we can, vertical and horizontal inheritance modes included, and cross-reference the information with that obtained from other genes. We call the resulting graphs *syntheses*, because they have both tree-like and web-like parts [21]. In doing so, we reckon as the supertree users, consensus makers or concatenation advocates do that recognizing the vertical backbone of a synthesis graph is an important part of the task, but nevertheless, just a part of the ultimate goal of phylogenetics.

### Using phylogenetic tree comparison to suggest LGT

This exercise requires an accurate identification of LGT. Many methods researching similarity/dissimilarity among genes exist to assess the occurrence of LGT [5,8,22], with varying reliability. Koski and Golding [23] showed that best BLAST hits do not necessarily represent the closest sequence relatives, casting doubt on the reliability of BLAST based approaches (at least when done naively). While Daubin et al. [24] showed that, in the bacterial realm at least, results from genome content and compositional approaches to detect LGT cannot always be taken as evidence for genetic exchange. In fact, it seems that phylogenetic analysis, even if it is more time consuming and might fail to detect transfers between more closely related organisms [5], is still one of the most reliable ways to investigate LGT [25]. Indeed, observing a close and robust phylogenetic relationship between gene sequences of distantly related organisms to the exclusion of gene sequences of more closely related organisms likely indicates LGT [26]. Alternative explanations, still possible *a priori*, would be artifacts of tree reconstruction, or complicated cases of reciprocal gene loss.

Highlighting conflicting signal between presumably correct gene trees is necessary in identifying LGT, although tree comparison is not trivial. Broad comparisons can be conducted between pairs of trees to determine if they are significantly different [15,27]. For instance, the incompatibility of different topologies can be assessed by an AU test for a given gene, using maximum likelihood estimates [28]. However, such global comparisons only indicate that there is a disagreement between the history of genes, not what this disagreement is. An alternative pruning of selected taxa and subsequent statistical tests of incongruency [29-31] can be attempted to identify the species misplaced in one of the two trees, considering one tree as a reference. Species to be pruned are generally chosen after an observation of bootstrap support values [32], as those robustly located in different parts of the trees under comparison. If the incongruence persists after these taxa have been removed, those species are then generally not considered responsible for the incompatibility between the trees. Conversely, if the pruning of species with significantly different locations in the trees eliminates incongruence, then these species could be considered as recipients of LGT, if no artifact is otherwise suspected of causing their odd placement in one of the topologies. In practice, this pruning approach is generally time-consuming and inefficient, due to the high number of possible pruning combinations, exacerbated by a possible misinterpretation of results due to taxon-sampling effects.

Recently, Addario-Berry *et al. *[33] implemented a promising comparison between a reference tree and a gene tree, overcoming some of these difficulties. Interestingly, their program posits lateral transfer schemes and scenarios to rationalize the differences between trees. These evolutionary scenarios, based on LGT, provide an explicit and accurate description of possible transfer events. In addition to the identification of a recipient species, they suggest a donor species, in the reference tree or outside of it. To do so, they estimate the minimum number of transfers necessary to explain disagreements between the pair of trees. This is done using the following criterion: since A and B are siblings in the gene tree, either the ancestral gene AB must have been present in the last common ancestor of A and B in the species tree or a lateral transfer event has occurred from the A lineage to the B lineage (or vice versa). At present, this method compares only one gene tree against the reference tree, and only if they include exactly the same taxa. In addition, it works only for rooted directed trees, which unfortunately limits its application. Notably, the requirement of strictly bifurcating trees forbids the collapse of unsupported nodes, thus greatly exaggerating the difference between trees.

In this paper, we revisit Addario-Berry *et al.*'s search for evolutionary scenarios to describe LGT but also insist on the detection of common vertical features among trees. We propose a pair of programs for dealing with multiple phylogenetic tree comparison, against a given reference tree. These trees may (or may not) differ in their species content, topological relationships, label positions, bootstrap support, and branch lengths.

## Implementation

### Horizstory

Comparing trees for two different genes amounts to exchanging branches within the first genes' tree (editing it) to match another's, and assessing the relative likelihood of this scenario. The topological comparison of phylogenetic trees is inherently difficult, as the number of possible edit paths (where one rearranges the branches of one tree to match another's) increases rapidly with the number of differences between trees. Where edits are individually independent, they may furthermore occur in any order within an edit path (evolutionary scenario), thus exploding their number factorially. Without resorting to heuristics and its inherent approximations, and without constraining the types of possible edits (apart from those that are biologically impossible: LGT with one's ancestor), we are therefore limited to comparing trees that are relatively similar. However, if one abandons the constraint of assuming that trees are fully resolved [33], which is most often not the case, then many apparent differences disappear and the problem becomes more tractable. One should be concerned with explaining only robust differences between trees, which can be determined by bootstrap support, for example, in ML phylogenies.

It is also important to compare the right trees, which amounts to a wise choice of reference tree against which test trees (typically gene-based trees) are compared. A good reference tree may be one that minimizes the overall number of differences it finds when matched with a variety of test trees; thus references based on concatenated sequences, supertrees, or genomic phylogenies should be most suitable for this purpose. Still, one might be uncertain about the exact choice of reference tree (or its optimal rooting), where alternatives are equally attractive. One could then use each candidate reference tree in turn as a reference against the set of test trees, and measure the extent of LGT predicted with each choice. This may suggest that one reference tree is better than another in this context, a result interesting on its own with respect to the issue of organismal phylogeny and the "true" tree.

Our approach to the comparison of phylogenetic trees, with the purpose of detecting lateral gene transfer (LGT) as well as determining the degree of concordance of vertical inheritance relationships, makes use of a recursive procedure of consolidation and rearrangement. Consolidation involves the simplification of identical topological features (vertical inheritance relationships) by collapsing such features [for example, a triplet-taxon relationship (("A","B"),"C") in common to both trees is collapsed to the single-taxon "((A,B),C)"]. This is followed in a second step by the collapse of compatible topological features. A compatible feature is, for example, a relationship of (("A","B"),"C") in the reference tree and an unresolved relationship of ("A","B","C") in the candidate tree, leading to " [A,B],C]". Compatible features do not necessarily support vertical relationships, but neither do they provide evidence for lateral gene transfer. Once the pair of trees to be compared is thus simplified, each of the candidate tree's leaves is moved to every alternative node in its tree in turn, with each move being tested by consolidation. Where simplification is possible (where the topologies can further converge), the move is productive and launches another pathway of rearrangement, where further rearrangements and simplifications are tried until the pair of trees can converge to identity, or until the pathway is abandoned. A pathway is abandoned, if, for example, it would require more steps than another pathway already explored.

We suppose that a rearrangement that can bring a pair of trees closer to one another topologically, is equivalent to "undoing" an event of lateral gene transfer. The position from which a taxon had to be moved in order to make the trees more similar is taken to be the LGT donor, whereas the taxon being moved is then the recipient. Given that rearrangement reconstitutes reference topologies (vertical inheritance relationships), presumed LGT targets are disqualified from suggesting such relationships by being pruned from the trees prior to the next recursive rearrangement.

Last but not least, rearrangement pathways must go to completion in order to be reported, resulting in full convergence of the pair of trees, but some edit distances are shorter than others, and suggest a smaller number of LGT events. For reasons of evolutionary parsimony as well as computational economy, this conservative route should be preferred.

Our scenarios can include different kinds of LGT events. Events of LGT may be nested, or otherwise intertwined, needing reversal by multiple rearrangements. The clade founded by an LGT donor may have subsequently had its species membership obfuscated by later exchanges of genetic material, yielding an unnatural assemblage of nomenclatural tags (species labels) in a presumed lineage. We distinguish such intermediary groupings in our output using an asterisk, indicating an ambiguity in deducing the root taxon, that being the actual organism that served as LGT donor. We also found it necessary to indicate (using a prime mark) when an LGT event cannot be attributed directly to a clade found in the reference tree, but rather to a phantom sister of that clade. This signals a 'basal transfer', which is observed when a taxon migrates out of its own clade to sit just outside of that clade in the candidate tree. Since LGT cannot occur with one's ancestor, the best explanation in a context of LGT is that the taxon received genetic material from a sister clade which happens not to be represented among the taxa in the dataset under investigation. Other nomenclatural marks in our program's output, mentioned above, include the parenthesis indicating an identical topological feature, and the square bracket indicating a compatible topological feature. The program's output thus consists of a list of the rearrangement pathways and the consequently deduced vertical and lateral (LGT) features. The frequencies of these features are also summarized in the output for each the pair of trees, and a global summary is presented for all pairs of trees analyzed.

For various reasons, a user might have a collection of trees to compare that include different taxa, or a user might more generally wish to exclude specific taxa from certain individual phylogenetic analyses. Where trees have different complements of taxa, pruning of taxa outside of the intersection of the two sets is done automatically. Where a pair of trees includes common taxa that the user wishes to exclude from analysis explicitly, he or she need only supply files listing which taxa should be excluded, either globally (applied to the reference tree and all of the candidate trees), or locally (applied to the reference tree and a candidate tree on a one-by-one basis). Pruning might be prescribed if, for example, one suspects causes other than LGT to be responsible for some of the differences between a pair of trees, such as long branch attraction. Absence of such files indicates that no pruning is desired.

We have successfully tested Horizstory with many different sets of real data, including hundreds of trees with 13 species and several dozen trees with over 27 species. Simple and nested LGTs simulated by manual modifications of a reference tree were also correctly reported in the scenarios.

### Lumbermill

**Figure 2:**
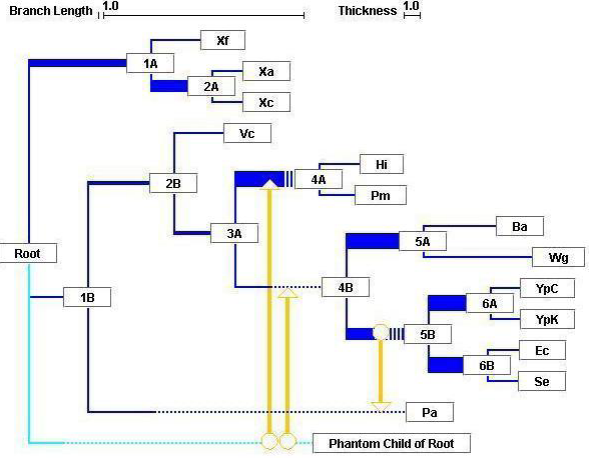
Synthesis diagram. The vertical-inheritance backbone representing the input reference tree is shown in dark blue, with the line thickness of an internal branch corresponding to the frequency of its support across the whole dataset. Putative LGT events are in orange, connecting donors (circles) with recipients (arrowheads); where there are multiple possible donor candidates, these converge onto a double arrowhead (see text). Where the apparent donor of a gene falls outside of the taxa included in the analysis, one is created as a basal group taxon, indicated in light blue. In order to avoid graphical congestion, branches in the tree may be artificially extended, as dotted segments. Colours are editable, and links are interactive. Clicking on node 3A, for example, displays the following message: 3A: set006r, branch length:0.03755, thickness:0.25, files supported: mvin.tre:2/2, where support for the node is 2/2 for mvin.tre (both edit paths support the node), and 0 (unsupported) for hypoprot.tre, biob.tre and n6methylase.tre. The segment's thickness, therefore, is simply (2/2)/4.

Lumbermill is a phylogeny editor, written in Java, for drawing trees and syntheses, using Horizstory output as input. It realizes our notion of synthesis (see Fig. 2) by first representing a vertical phylogram as its backbone. Line thickness is drawn in proportion to the percentage of genes supporting a given grouping; common patterns of vertical inheritance are thus easy to identify. (The assumption here is that over the short term, vertical inheritance is the dominant pattern). Overlaid onto this backbone are links that represent presumed LGTs, completing the synthesis.

The images trees or syntheses are highly editable, allowing the user to change fonts and line colours to provide a customized view of the data. The order of a node's descendants can be swapped. The backbone can be rerooted on another node or even unrooted. To help describe events in the synthesis, nodes are labeled numerically by distance from the root then alphabetically from top to bottom. In addition to this, each link is interactive, and when clicked on, displays information such as the proportion of genes inherited along this link, and their names.

Often, multiple equally parsimonious LGT scenarios are proposed by Horizstory in order to explain differences between a given gene tree and the reference topology. Lumbermill allows the user to restrict the display of individual LGT events to those suggested by a specified minimum fraction of the scenarios, such as 1.0 (strict consensus) or values greater than 0.5 (majority rule). One can also elect to omit the display of specific LGTs for some genes in Lumbermill, such as when the conflicting signal involving this gene is found to be due to some cause other than lateral gene transfer.

Putative LGT events are drawn as arrows originating from a donor (indicated by a circle) and terminating at a recipient. Since the exact time at which a transfer occurred cannot be determined, the relative order of multiple arrows on any given segment is irrelevant, as is the position of an arrow on the segment. In order to avoid clutter in busy regions of the tree, we chose to extend segments to provide more room. Such artificially extended segments are drawn as dotted lines so as not to confuse them with actual branch lengths (solid lines).

When genes have apparently been inherited from a taxon missing from the reference tree, we insert a basal group in the tree where appropriate. This donor, a contemporary clade absent from the current dataset and that may or may not have since gone extinct, collects all of the LGT events originating from outside of a represented clade. It is meant as a convenient catchall, and where multiple LGTs appear to originate from such a given basal group, it is understood that different donors may actually have contributed genes independently.

Our method allows for nested LGT scenarios, where a compound donor in the evolutionary scenario for a gene is not represented as an organismal clade in the reference tree. In this case, one cannot therefore point to an actual donor for the gene at this intermediary step in the scenario, since its identity is ambiguous, appearing to parent species from different clades. Lumbermill deals with such organismal assemblages by indicating several candidate donors all leading to the same target. It is assumed that the LGT event in question involved a single donor parenting one or more species in this assemblage, or when a basal group is also indicated, a single donor related to the parent of one or more of the species in the assemblage. These LGT involving such intermediate assemblages are represented using a double-headed arrow.

## Results

**Figure 1:**
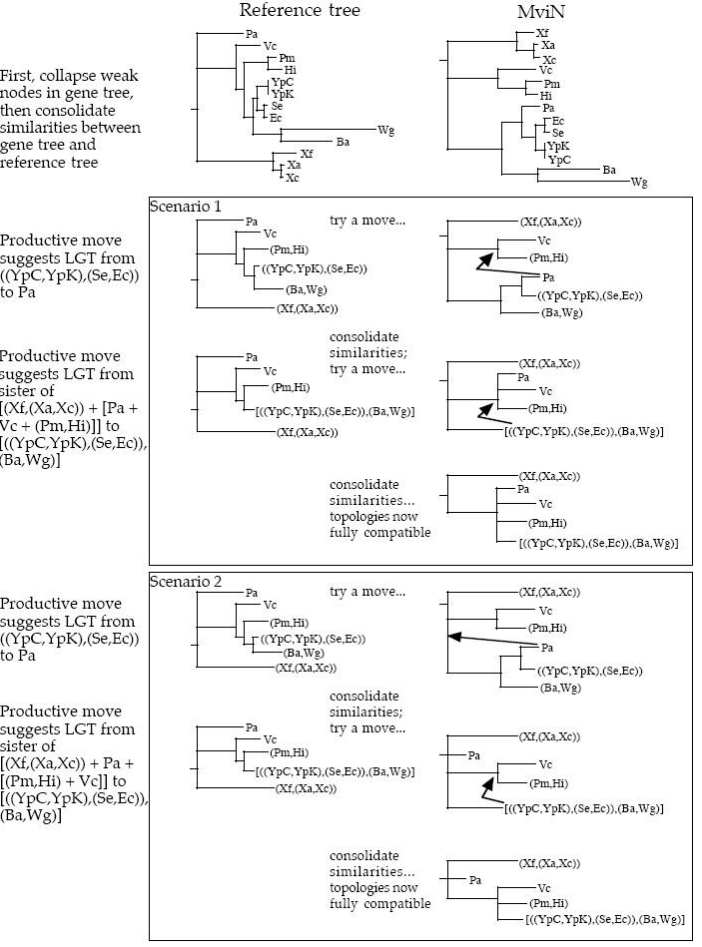
Step-by-step example of the consolidation and rearrangement method of Horizstory. Where indicated as resolved, nodes were supported by a bootstrap value better than 80%. Two minimal scenarios were found in the analysis of the MviN tree shown in this example, both suggesting an equivalent pair of lateral gene transfer events (as illustrated in Lumbermill, Figure 2). Other (longer or unproductive) scenarios are not shown. A productive move is one where the gene tree gains further similarity to the reference tree, permitting further consolidation (see text). Ba, *Buchnera aphidicola*; Ec, *Escherichia coli*; Hi, *Haemophilus influenzae*; Pa, *Pseudomonas aeruginosa*; Pm, *Pasteurella multocida*; Se, *Salmonella enterica*; Vc, *Vibrio cholerae*; Wg, *Wigglesworthia glossinidia*; Xa, *Xanthomonas axonopodis*; Xc, *Xanthomonas campestris*; Xf, *Xylella fastidiosa*; YpC, *Yersinia pestis *CO92; YpK, *Yersinia pestis *KIM.

We provide an example for four genes of the gamma-proteobacterial core compared with the gamma-proteobacterial species tree [34], deduced from a simultaneous study of 205 genes by Lerat *et al*. (Our previous critique of this tree [21] was focused on its statistical support, not its biological reasonableness). In order to illustrate the workings of Horizstory on a simple example, Figure 1 displays the analysis of a tree for the virulence factor MviN against this proposed gamma-proteobacterial species tree. The two steps required to transform the MviN tree into one compatible with the reference tree are in fact equivalent in the two scenarios shown (differing only in hypothetical intermediates), and can thus be represented more simply in the user-interface layer of our software, Lumbermill, described above.

Among their 205 markers, Lerat *et al*. [34] identified two proteins, the virulence factor MviN and the biotin synthase BioB, for which the position of *Pseudomonas aeruginosa *conflicted with the species tree. Our example (Fig. 2) includes these two genes, with two more randomly chosen from among the remaining 203 of Lerat *et al.*'s set: a putative ORF and N6-adenine-specific methylase. It shows that some but not all recent divergences are reasonably supported by the set of four genes that contributed to this synthesis (*e.g.*, the monophyly of the endosymbionts *Buchnera aphidicola *and *Wigglesworthia glossinidia *has a thick link), whereas deeper nodes are generally not so well supported, suggesting that this reference tree is not robust. This is consistent with the fact that mutational saturation of molecular sequences contributes to the erasure of phylogenetic information as time progresses [35], and several reports indicate that even with large amounts of data, the resolution of deep phylogenies will continue to be elusive [36,37].

Lumbermill displays both the support for a clade as well as the putative shuffling of genes with other clades, and can therefore begin to address this issue. For instance, Figs. 1 and 2 show that the ancestor of enterobacteria (represented by *Y. pestis*, *E. coli*, *S. enterica*, *B. aphidicola *and *W. glossinidia*) acquired its copy of MviN from a sister group of the gamma-proteobacteria by LGT, and subsequently the non-endosymbiotic enterobacteria donated this gene to *P. aeruginosa*. Fig. 2 also shows that a copy of BioB was laterally transferred from a sister group of the gamma-proteobacteria to the ancestor of *P. multocida *and *H. influenzae*.

**Figure 3:**
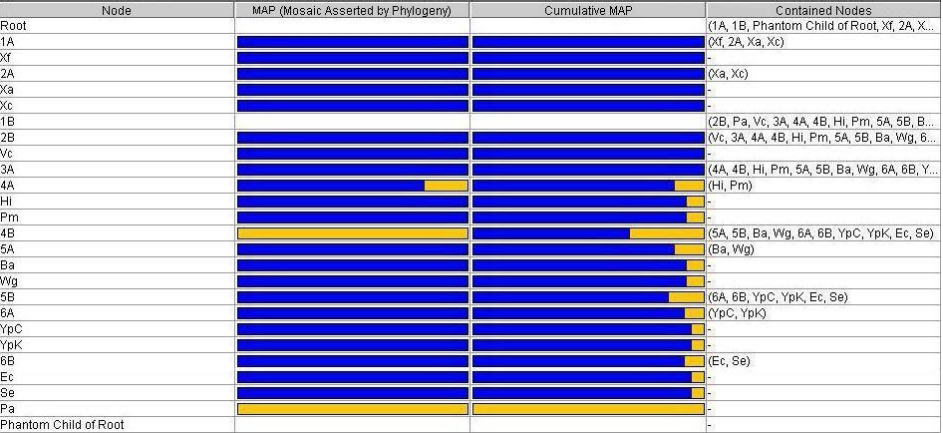
Estimate of genomic mosaicism. This application screenshot displays a table indicating the degree of vertical and of horizontal inheritance inferred for each node in the synthesis (see Fig. 2). The middle two columns of the table summarize the distribution of vertical (blue) and of lateral (orange) support for the segment immediately preceding each node, and the vertical and lateral support for the whole string of segments leading to a particular node, respectively. For example, *B. aphidicola *shows a 14% degree of accumulated mosaicism since the tree's root (from the small sample of genes used in this analysis), but no recent mosaicism (in the segment preceding its divergence from *W. glossinidia*, node 5A). Numerical values for the proportions are given by clicking on a bar. Node 1B is left entirely blank for want of phylogenetic evidence.

Many estimates, described in the help file of Lumbermill (available online), are also implemented in Lumbermill to help investigate the distribution of the phylogenetic signal in the synthesis, to get information about the processes of LGT and a rough description of genetic mosaicism by node, as presented in Fig. 3. This last feature of Lumbermill addresses concerns raised by important publications [38-40], describing organisms as chimerae. Although a tree-like framework can still be appropriate to relate species for which no mosaicism is detected, a web [2] or a synthesis such as ours is required where mosaicism is evident. Fig. 3 displays the relative proportion of vertical inheritance (in blue) and of lateral transfer (in orange), phylogenetically assessed for each node of the synthesis. The information is displayed in two columns: the first represents lateral transfer occurring since the previous node only, while the second shows a cumulative estimate of all LGTs since the origin. Such summaries can address the issue relating phylogenetic distance with propensity for exchange, known to exist within "species" [41,42] and postulated for larger clades [43,44].

## Conclusion

Horizstory and Lumbermill constitute a pair of phylogenetic tools to compare multiple trees, and to display this comparison in a useful and flexible format. The software is freely available to the community and downloadable from , and it will continue to be built upon in order to add further summary analyses. Already, we believe that they provide important information to the evolutionary biologist such as the frequency and direction of putative LGT events. We also gain information on the number of lateral exchanges undergone by each gene, the uniqueness of the LGT event (a unique event generates a thin horizontal link) or the fact that some species exchanged multiple genes on a regular basis (thick horizontal lines). Finally, we can assess the robustness of a tree's backbone. One could use these analyses in order to explore the evolution of phylogenetic signal and the relative power of phylogenetic methods to solve a given taxonomic issue. By proposing specific LGT events, our system may furthermore bracket the temporal coexistence of lineages, given that LGT can only occur between contemporary species. Otherwise, one cannot know much about the temporal coincidence of anything but extant species, where fossil evidence is lacking. This may be combined with ecological and biogeochemical hypotheses, and thereby help to understand the propagation of biological innovation throughout evolutionary time.

## Availability and requirements

*Project name: Horizstory and Lumbermill

*Project home page:  (all programs and test files alternatively available from EB upon request)

*Operating system(s): Platform independent (Horizstory requires a command line interface)

*Programming languages: Horizstory – C++, Lumbermill – Java

*Other requirements: C++ Compiler (Horizstory), Java 1.4.0 or higher (Lumbermill)

*License: Horizstory – GNU GPL, Lumbermill – none

*Any restrictions to use by non-academics: Horizstory – none, Lumbermill – none

note: Horizstory was designed and tested using gcc 3.3, available at 

### Application details

Whereas Lumbermill has an intuitive graphical user interface, Horizstory is a command-line C++ program whose options we describe below. Its signature is:

HorizStory -f listOfTreeFiles -r indexOfReferenceTree -m minimumBootstrap [-t fractionThreshold] [-bias twoLetterCode] [-s timeLimitPerTreeInSeconds] [-v]

where square brackets indicate optional parameters; the default fraction threshold is 0.0, the default bias is none, the default time limit is 86400 s (1 day), and the default verbose mode (the -v option) is false.

-f: Names a file that first specifies the number of entries (*e.g.*, 7), then specifies that number of file names, representing a reference tree and trees to be tested against it, in any order. Non-reference trees must include bootstrap values for each node (or they are considered to be equal to 100%), but the reference tree need not include such information. Our method requires trees to be rooted, but can deal with multifurcated trees.

-r: The index, within the listOfTreeFiles, of the reference tree's file. The reference tree is assumed to be topologically resolved and rooted; where the user wishes an unresolved feature (a multifurcation) within the reference tree, a branch length of 0.0 can be specified. Bootstrap values may or may not be present in the tree; if present they are ignored unless 0, which again serves to collapse a node.

-m: Branch lengths of 0.0 in a candidate tree are automatically unresolved; otherwise bootstrap values are compared with this integer parameter in order to decide whether or not the node should be collapsed. For example, a weak feature (A,B) below threshold in ((A,B),C) collapses to (A,B,C).

-t: When trees are quite different, many pathways of rearrangement might be found that can transform a candidate tree into a topology compatible with the reference tree. In some of these pathways, exotic LGT events might be proposed that are not proposed by the majority of other pathways. This parameter, therefore, allows one to limit the summary output of the program to LGT that are suggested by at least a certain fraction of the pathways, *e.g. *0.1.

-bias: One may wish to minimize or to maximize proposed events of LGT involving nested groups or basal groups. The analysis is first done to minimize the length of a rearrangement scenario, but then the output can be filtered by one of the following options if desired.

n: consider paths with the minimum number of nested groups, from among the set of shortest edit paths.

np: consider paths with the minimum number of nested groups, and of those, with the minimum number of phantom sisters.

nP: consider paths with the minimum number of nested groups, and of those, with the maximum number of phantom sisters.

N: consider paths with the maximum number of nested groups.

...and so on (Np, NP, p, pn, pN, P, Pn and PN).

-s: The user may specify a time limit for the analysis of each tree, in seconds. Tests on pairs of randomly generated trees indicate that processing time is exponential, O(c^n^), with the number of differences in the trees after initial consolidation, n, with c = 6.1, and on our test system (a 2.0-GHz PowerPC G5), with O being approximately 1.5 μs. Where a pair of trees must be compared, but the required time is prohibitive, the user may opt to increase the minimum bootstrap (-m) requirement, in order to compare only the most resolved portions of the trees.

-v: When specified, this parameter enables verbose output to the terminal window. It does not affect the output printed to file.

"Pruning files" are given the identical name of the referenceTree, but appended with ".pru", for global prunings, or are given the identical name of a candidate tree (appended with ".pru") for local prunings. The file specifies the number of taxa to be pruned, followed by that number of taxon names.

## Authors' contributions

EB originated the study and provided critical feedback to DM, who implemented Lumbermill, and to RLC, who implemented Horizstory. WFD contributed to the writing of the manuscript.
